# Dual redundant sequencing strategy: Full‐length gene characterisation of 1056 novel and confirmatory HLA alleles

**DOI:** 10.1111/tan.13057

**Published:** 2017-05-25

**Authors:** V. Albrecht, C. Zweiniger, V. Surendranath, K. Lang, G. Schöfl, A. Dahl, S. Winkler, V. Lange, I. Böhme, A. H. Schmidt

**Affiliations:** ^1^ DKMS Life Science Lab Dresden Germany; ^2^ Deep Sequencing Group CRTD – Center for Regenerative Therapies Dresden Dresden Germany; ^3^ DNA Sequencing, Max Planck Institute of Molecular Cell Biology and Genetics Dresden Germany; ^4^ DKMS Tübingen Germany

**Keywords:** full‐length gene sequencing, HLA typing, NGS, novel HLA alleles, PacBio

## Abstract

The high‐throughput department of DKMS Life Science Lab encounters novel human leukocyte antigen (HLA) alleles on a daily basis. To characterise these alleles, we have developed a system to sequence the whole gene from 5′‐ to 3′‐UTR for the HLA loci A, B, C, DQB1 and DPB1 for submission to the European Molecular Biology Laboratory – European Nucleotide Archive (EMBL‐ENA) and the IPD‐IMGT/HLA Database. Our workflow is based on a dual redundant sequencing strategy. Using shotgun sequencing on an Illumina MiSeq instrument and single molecule real‐time (SMRT) sequencing on a PacBio RS II instrument, we are able to achieve highly accurate HLA full‐length consensus sequences. Remaining conflicts are resolved using the R package DR2S (Dual Redundant Reference Sequencing). Given the relatively high throughput of this strategy, we have developed the semi‐automated web service TypeLoader, to aid in the submission of sequences to the EMBL‐ENA and the IPD‐IMGT/HLA Database. In the IPD‐IMGT/HLA Database release 3.24.0 (April 2016; prior to the submission of the sequences described here), only 5.2% of all known HLA alleles have been fully characterised together with intronic and UTR sequences. So far, we have applied our strategy to characterise and submit 1056 HLA alleles, thereby more than doubling the number of fully characterised alleles. Given the increasing application of next generation sequencing (NGS) for full gene characterisation in clinical practice, extending the HLA database concomitantly is highly desirable. Therefore, we propose this dual redundant sequencing strategy as a workflow for submission of novel full‐length alleles and characterisation of sequences that are as yet incomplete. This would help to mitigate the predominance of partially known alleles in the database.

## 1 INTRODUCTION

The human leukocyte antigen (HLA) system, also known as human major histocompatibility complex (MHC), is located on chromosome 6 at 6p21.3.^1^ The system plays a central role in immune response and disease associations. It impacts organ and stem cell transplantation as the major genetic determinant of graft acceptance.^2^ The 6 classical HLA genes HLA‐A, ‐B and ‐C (class I) and HLA‐DRB1, ‐DQB1 and ‐DPB1 (class II) residing in the system are amongst the most polymorphic genes in humans.^3^

For the last 18 years, the IPD‐IMGT/HLA Database has provided a centralised publicly accessible database to collect and report the hyperpolymorphic allele sequences of the HLA genes.^4^, ^5^ Every year hundreds of novel HLA alleles are described and submitted resulting in a rapidly growing database. By January 2017, over 16 000 HLA alleles had already been named and characterised (http://www.ebi.ac.uk/ipd/imgt/hla/stats.html). However, for a large proportion of these alleles only a small part of the sequence is known. It is still a common practice in many laboratories and scientific institutions to submit only the sequence information for the core exons 2 and 3, which has led to a predominance of partially known alleles in the database and limited information on the diversity of intronic sequences. Thus, in the IPD‐IMGT/HLA Database release 3.24.0 (April 2016), prior to the submission of the bulk of the sequences described here, only 5.2% of all known HLA alleles had been fully characterised if we consider as fully characterised those alleles for which the complete sequence including all introns and both UTRs is known. Until recently, the limited representation of most alleles matched the state‐of‐the‐art technologies for HLA characterisation.

Next generation sequencing (NGS) technologies are now beginning to be adopted in clinical settings.^6^ The full‐length sequence characterisation by NGS provides opportunities to identify sequence mismatches outside of the antigen‐presenting region and to catch rare or even so far undocumented null alleles. While the relevance of sequence information pertaining to the peptide binding groove is well established,^7^ the clinical impact of other regions of HLA genes is largely unknown. It would appear short‐sighted, however, to dismiss their clinical potential based on our current understanding. Providing this information to the medical community will permit future interrogation of the impact of introns, expanded exons and other gene regulatory sequences on clinical outcomes in transplantation and may ultimately help to make possible much more precisely targeted treatments.^8^ However, at present, the vast majority of the alleles in the IPD‐IMGT/HLA Database are only partially characterised. This also entails that allele matching processes are inherently biased towards partially known alleles, given that their reduced sequence space has potentially fewer differences relative to full‐length alleles. The capability of state‐of‐the‐art NGS platforms to generate full‐length allele sequences in a cost‐effective and timely fashion may help to alleviate the predominance of partially known alleles in the IPD‐IMGT/HLA Database. For this purpose, we propose a dual redundant sequencing strategy.

The most successfully and widely adopted NGS technology is currently sequencing‐by‐synthesis (SBS) on Illumina platforms (Illumina, San Diego, California).^9^ For example, an Illumina MiSeq instrument generates very accurate data at low costs in a high‐throughput fashion.^10^ Due to the limited read length achievable by SBS, large polymerase chain reaction (PCR) products, for example, those covering complete HLA genes, need to be fragmented into smaller pieces before sequencing. The disadvantage of such a shotgun approach is the subsequently required short‐read assembly process which potentially leads to loss of phase in heterozygous situations. By contrast, the PacBio System (Pacific Biosciences, Menlo Park, California) offers long read lengths in the order of 12 kb with the single molecule real‐time (SMRT) sequencing technology.^11^ This method provides a fast sample preparation and turnover rate as well as phased sequences.^12^ One of the limitations of SMRT sequencing is the relatively high error rate (>10%) at the individual read level.^13^, ^14^ Due to the largely random distribution of the errors, highly accurate consensus sequences can be obtained nevertheless. However, the high rate of insertion or deletion errors makes it difficult to accurately call homopolymer stretches and repetitive sequences.^14^ The complementary strengths of the Illumina SBS and Pacific Biosciences’ SMRT technology, in combination, lay the foundation for a propitious approach to generate highly accurate full‐length HLA allele sequences.

In this paper, we describe a workflow that has been specially developed for whole‐gene sequence characterisation for the submission of novel alleles and sequences that are as yet incomplete in the IPD‐IMGT/HLA Database. Each sample is analysed with SBS and SMRT sequencing independently to achieve highly accurate sequence characterisation. We report the full‐length characterisation of 1056 HLA alleles and their subsequent submission to the European Molecular Biology Laboratory – European Nucleotide Archive (EMBL‐ENA) and the IPD‐IMGT/HLA Database using the in‐house developed software TypeLoader (DKMS Life Science Lab, Dresden, Germany).^15^

## 2 MATERIAL AND METHODS

### 2.1 Samples

A total of 1056 whole blood or buccal swab samples were subjected to full‐length HLA gene characterisation. DNA was isolated using the chemagic DNA Blood/Swab Kit 150 special according to the manufacturer's instructions (PerkinElmer, Baesweiler, Germany). The samples were eluted in 10 mM Tris/HCL, pH 8.0. DNA concentration was measured with SYBR Green fluorescence (Biozym, Hessisch Oldendorf, Germany).

### 2.2 Polymerase chain reaction

Long‐range PCR was performed with primers located outside the UTR regions of the HLA genes. Between 4 and 400 ng genomic DNA was amplified with the GoTaq Long PCR (Promega, Madison, Wisconsin) kit. Class II amplification was performed with 4% DMSO. Two independent PCR reactions were performed on each sample for the 2 sequencing approaches, SMRT sequencing and shotgun sequencing, using primer mix concentrations of 0.08 and 0.8 μM, respectively. Both PCR primer sets targeted the same positions. Primers differed only by generic 5′ sequence extensions on the SMRT primers for the indexing PCR. An additional nested PCR reaction was performed for samples with weak amplification based on gel electrophoresis analysis. For the SMRT sequencing approach, the samples were barcoded/indexed by an additional PCR reaction with indexing primers (0.2 μM) using indices as suggested by Pacific Biosciences.^10^ All primers were obtained from metabion (metabion international, Martinsried, Germany) and TIB Molbiol (TIB Molbiol Syntheselabor, Berlin, Germany).

### 2.3 Library preparation for shotgun and SMRT sequencing

To fragment PCR products for shotgun sequencing on Illumina instruments, an adapted NEBNext dsDNA Fragmentase protocol was used (New England Biolabs, Frankfurt, Germany). PCR products containing 2.5 mM MgCl_2_ were treated with 0.125× NEBnext dsDNA Fragmentase at 37°C for 30 minutes. The samples were purified with 0.7× SPRIselect beads (Beckman Coulter, Brea, California). For library preparation the NEBnext Ultra DNA Library Prep Kit for Illumina was used in combination with NEBNext Multiplex Oligos for Illumina (both from New England Biolabs, Frankfurt, Germany). A total of 2 × 48 samples were pooled and purified using a ratio of 0.6:1 SPRIselect beads. For equimolar pooling, the samples were quantified by qPCR with an ECO Real‐Time PCR cycler (Illumina) using an in‐house qPCR mix and KAPA DNA standards for Illumina (KAPA Biosystems, Boston, Massachusetts). The barcoded amplicons were sequenced on an Illumina MiSeq instrument performing a 251 bp paired‐end sequencing run for 96 samples using MiSeq Reagent Kit v2. The MiSeq flow cell was loaded with a 7.5 pM library containing approximately 10% PhiX (MiSeq Reagent Preparation Guide).

For SMRT sequencing, 96 samples were pooled for each locus, except in the case of HLA‐DPB1, where the library was split into 2 pools containing 48 samples each. The library preparation was carried out according to manufacturer's instructions (Pacific Biosciences Template Preparation and Sequencing Guide). Libraries with novel alleles in HLA‐B, ‐DQB1 and ‐DPB1 were size selected with the BluePippin system using a 0.75% cartridge according to the BluePippin DNA Size Selection System Operations Manual (Sage Science, Beverly, Massachusetts). The samples were prepared according to Pacific Biosciences’ standard protocol and run on a PacBio RS II instrument with 80–100 pM “on plate” loading concentrations translating to 10–20 pM “on chip” loading concentrations per library.

### 2.4 Data analysis

Analysis of data was performed with the NGSengine software (GenDx, Utrecht, the Netherlands).^16^ The 2 different sequencing approaches were analysed separately and independently. The results were compared manually. In cases where the sequences obtained from shotgun SBS and SMRT sequencing contained any differences, the following rules were applied:

1. If the long‐read (SMRT) consensus sequences showed a deviation from the closest reference allele, which was not confirmed by the short‐read (SBS) consensus sequence, the SMRT sequence deviation was flagged as a sequencing error and the SBS consensus sequence was submitted.
2. If the SBS consensus sequence showed a deviation from the closest reference allele, which was not confirmed by the SMRT consensus sequence, the analysis for this sample was repeated.
3. If the whole‐gene SBS sequence was not phase‐defined and the SMRT data suffered from insertion/deletion errors, DR2S (Dual Redundant Reference Sequencing) was applied to derive an accurate consensus sequence.^17^

By applying these rules, we took advantage of the error correcting potential of the dual sequencing approach but avoided unnecessary repeats of sequencing reactions whenever a sequence difference was clearly identified as a sequencing error due to the limitations of the platform used. We developed the rule set based on the following observations and ideas:

- Most submitted novel alleles differ only in 1 or very few bases from the closest reference allele. Therefore, the likelihood that a sequencing artefact results in an artificial sequence deviation is much higher than the chance to revert a real difference back to the reference sequence. For example, given a novel class I gene with 4 differences to the closest reference, there are 4 positions for sequencing errors that may mask a true sequence difference (if the sequencing error leads to the matching base). However, there are 3400 positions where a sequencing error may introduce an artificial difference. If only 1 sequence is obtained, it cannot be determined which sequence differences are real and which are due to artefacts. However, if the second sequencing approach confirms the reference sequence the likelihood for an error is negligible given that sequencing errors occur at random.
- The long‐read SMRT sequencing data suffer from insertion/deletion errors.^14^ Given sufficient coverage, the consensus sequences overall have a very low error rate. However, an imbalance in insertion and deletion error rates in homopolymeric stretches and short tandem repeats may still sometimes lead to erroneous SMRT consensus sequences for such regions.^14^ If the reference and the SBS sequence agree, we therefore disregard such discrepancies in the SMRT data (rule 1). Adhering to this principle resolved most conflicts between sequencing data from the 2 approaches.
- Given sufficient coverage, the consensus quality of the SBS data was extremely high and we identified only very few cases where we had to apply rule 2. Because those cases were rare, they did not increase the work load markedly. In all those cases resequencing confirmed the reference/SMRT sequences and rejected the SBS data. Therefore, treating the SBS discordances in the same way as the SMRT data (rule 1) appears to be a viable option.
- DR2S creates 2 multiple alignment consensus sequences from allele‐separated SMRT reads. The short SBS reads are mapped against these consensus sequences to facilitate the correction of the remaining sequencing errors. By integrating the data of both sequencing technologies, DR2S delivers highly accurate sequences even for challenging sequence regions. DR2S was mostly applied for class II alleles which regularly exhibit sequence length variations in intronic regions and multiple sequence deviations from the reference alleles.

### 2.5 Submission

Data were submitted to the EMBL‐ENA and the IPD‐IMGT/HLA Database using TypeLoader (http://www.github.com/DKMS-LSL/typeloader).^15^ Only the locus with the potentially novel sequence was submitted for the dual redundant sequencing approach. For submission, the complete HLA types for the HLA‐A, ‐B and ‐DRB1 genes of the sample from which the novel allele was derived must be reported. We obtained this typing information directly from our routine high‐throughput workflow. Table 1 gives a short overview of the tools and databases used during this project.

**Table 1:** Tools and databases

Tools/databases	Description
NGSengine	Platform‐independent software for the high‐resolution identification of HLA alleles by NGS (http://www.gendx.com/ngs-engine-download).
DR2S	Dual Redundant Reference Sequencing (DR2S) is an R package designed to facilitate the generation of reliable, full‐length phase‐defined reference sequences for novel HLA alleles (https://github.com/gschofl/DR2S).
TypeLoader	Automatic EMBL‐ENA and IPD‐IMGT/HLA Database Upload Generator. This web service takes FASTA and GenDx generated XML files, processes them and automatically generates data for direct submission to the EMBL‐ENA and the IPD‐IMGT/HLA Database (https://github.com/DKMS-LSL/typeloader).
ENA	The EMBL‐ENA provides a comprehensive record of the world's nucleotide sequencing information, covering raw sequencing data, sequence assembly information and functional annotation (http://www.ebi.ac.uk/ena).
IPD‐IMGT/HLA Database	The IPD‐IMGT/HLA Database provides a specialist database for sequences of the MHC and includes the official sequences named by the WHO Nomenclature Committee for factors of the HLA System. The IPD‐IMGT/HLA Database is part of the international ImMunoGeneTics project (IMGT) (http://www.ebi.ac.uk/ipd/imgt/hla/).

EMBL‐ENA, European Molecular Biology Laboratory – European Nucleotide Archive; HLA, human leukocyte antigen; NGS, next generation sequencing; WHO, World Health Organization.

### 2.6 HLA typing by serology

The microlymphocytotoxicity test Lymphotype HLA ABC 72 (Bio‐Rad Medical Diagnostics, Dreieich, Germany) was applied for serological confirmation of null alleles.

## 3 RESULTS

### 3.1 Workflow

**Figure 1:**
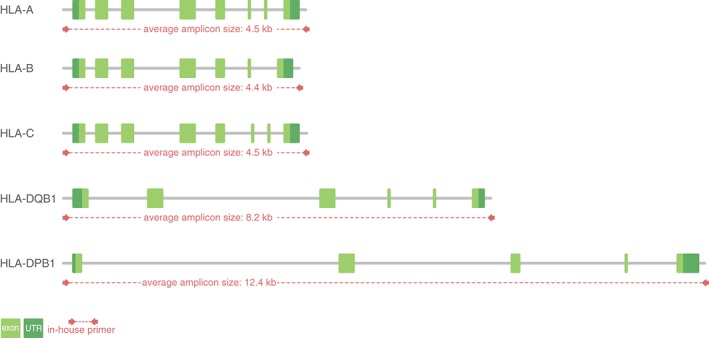
Genomic organisation of the human leukocyte antigen (HLA) loci. The class II alleles are about 2 to 3 times the length of the class I alleles. All primers applied during this project are located outside the UTR regions

The increasing sample throughput in the high‐throughput department of DKMS Life Science Lab has seen a concomitant increase in the identification of novel alleles. At the time of analysis, approximately 13 000 samples had been identified, which harbour sequences without corresponding reference sequences in the IPD‐IMGT/HLA Database. This includes 10 850 samples with novel sequences in the HLA loci A, B, C, DQB1 and DPB1 (Table 2) and 1915 samples with novel sequences in the HLA‐DRB1 locus (enquiry date: July 26, 2016). Therefore, we have developed a workflow to sequence the whole gene from 5′ to 3′UTR for the HLA loci A, B, C, DQB1 and DPB1 (Figure 1). A workflow to process HLA‐DRB1 is still in development.

**Table 2:** Breakdown of whole‐gene sequence submissions from DKMS Life Science Lab to IPD‐IMGT/HLA release 3.27.0

HLA locus	Whole‐gene sequence submissions				
	Distinct				
	Novel alleles	Extended sequences	Total	Confirmatory	Total
A	163	72	235	41	276
B	98	54	152	10	162
C	282	120	402	58	460
DQB1	59	43	102	49	151
DPB1	4	3	7	0	7
Total	606	292	898	158	1,056

HLA, human leukocyte antigen.

**Figure 2:**
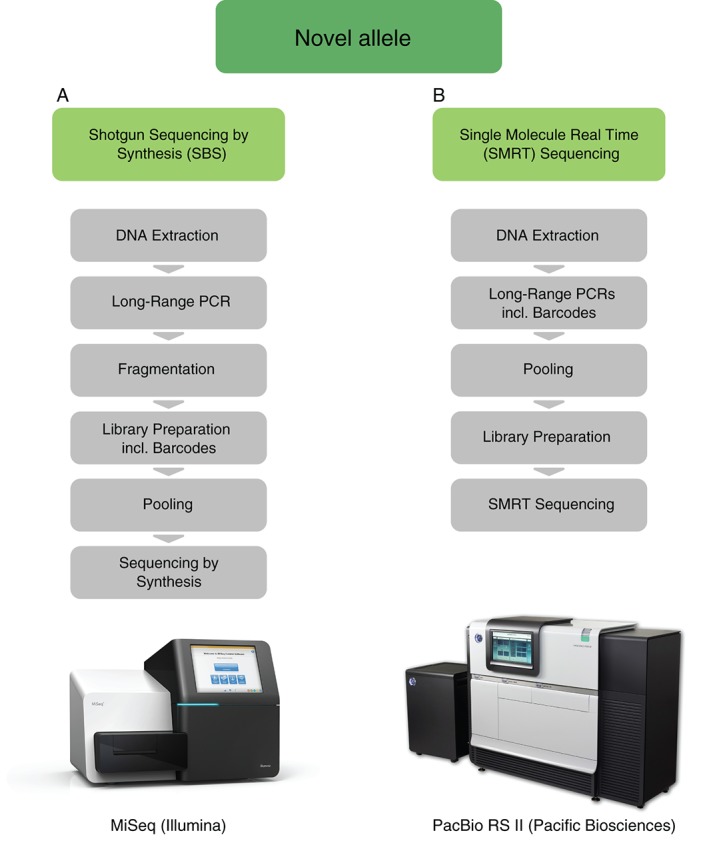
Workflow for full‐length HLA gene characterisation showing the dual redundant sequencing strategy using the MiSeq and PacBio RS II platforms. A, MiSeq requires a fragmentation step owing to its inability to completely sequence molecule fragments longer than 600 bp; barcodes are attached during library preparation. B, Barcoding is carried out as a part of the polymerase chain reaction (PCR)

The workflow allows characterising highly accurate whole‐gene consensus sequences by using 2 different sequencing technologies: shotgun SBS sequencing on an Illumina MiSeq instrument and SMRT sequencing on a PacBio RS II (Figure 2). This dual redundant sequencing strategy is applied to each sample. Two independent PCR reactions are performed to facilitate identifying PCR artefacts. The sequence data is analysed with the NGSengine software from GenDx and the in‐house software DR2S (https://github.com/gschofl/DR2S). For bulk submission of novel sequences to the EMBL‐ENA and the IPD‐IMGT/HLA Database, we have designed the TypeLoader software.

### 3.2 Necessity of the dual redundant sequencing strategy

It is a general requirement for the submission of sequences based on PCR‐amplified material to the IPD‐IMGT/HLA Database that the submitted sequences should be based on 2 independent PCR reactions. This requirement aims to prevent the submission and naming of sequences containing errors due to unspecific PCR artefacts. However, it has also become clear that often 1 whole‐gene sequencing method alone is not sufficiently reliable to define extended genomic sequence regions.^17^, ^18^ In the absence of a highly similar full‐length reference allele (*de novo* sequencing), the correct phasing of 2 heterozygous positions separated by large distances may become problematic. On the other hand, inherent systematic biases due to the sequencing strategy applied can lead to falsely characterised sequences. Therefore, we perform not only 2 independent PCR reactions but also analyse these reactions on 2 independent sequencing systems based on complementary technologies.

**Figure 3:**
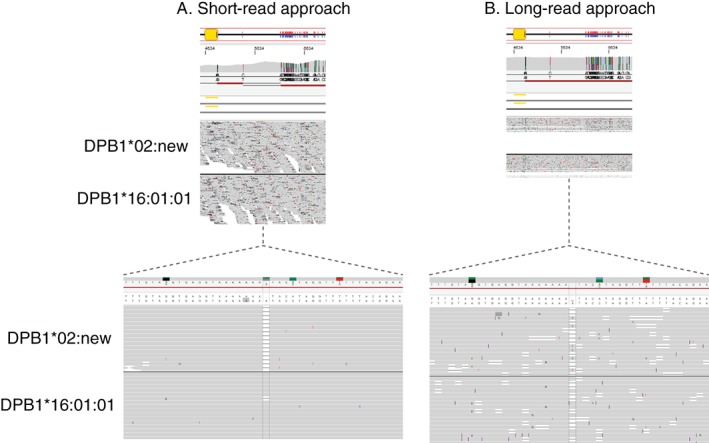
Limitations of each sequencing method. A, Illustration of the inability of accurate phasing using short sequencing‐by‐synthesis (SBS) reads (DPB1*02:new represents a yet unnamed novel allele). B, Inability to call homopolymer consensus sequences accurately due to a high insertion/deletion sequencing error rate with long single molecule real‐time (SMRT) reads

An example (Figure 3) shows how this strategy allows the resolution of typical sequencing challenges:

When 2 heterozygous positions are separated by a distance of more than 1000 bp, the short‐read approach is not able to phase the 2 sequences. The loss of phasing is shown by a broken red line. This is caused by the limiting maximum length (~1000 bp) of fragments which effectively form clusters on current Illumina MiSeq instruments. In contrast, SMRT sequencing generates contiguous long reads and therefore phase‐resolves both alleles. In comparison to the short‐read method, however, SMRT sequencing is prone to sequencing errors, especially in homopolymeric and repetitive sequence regions.^11^, ^13^ In this particular example (Figure 3), a high rate of insertion/deletion errors results in a situation where the difference in the length of a poly‐A‐stretch between the 2 alleles remains inconclusive. However, the SBS data clearly indicate that just 1 allele has a deletion while the other is in concordance with the reference sequence. In this case, the application of SMRT sequencing technology alone would ultimately have led to an incorrect consensus sequence call and thus to an erroneous submission to the IPD‐IMGT/HLA Database. In order to obtain a highly accurate sequence for this sample, DR2S was applied. This in‐house software uses long reads initially to cluster reads derived from the 2 alleles of a heterozygous sample and to construct preliminary haplotype consensus sequences. Subsequently, DR2S maps short reads against these consensus sequences, thereby identifying and correcting inconsistencies.^17^ The integration of the data from both technologies yields reference quality sequence data.

### 3.3 Characterised alleles

DKMS Life Science Lab has genotyped more than 2.7 million samples for 6 HLA loci using a high‐throughput short amplicon NGS approach^10^, ^19^ and almost 500 000 samples using Sanger based HLA typing. During this routine donor registry typing, we have identified 10 850 samples with sequences in the HLA loci A, B, C, DQB1 or DPB1 that were not described at the time of analysis in the IPD‐IMGT/HLA Database. A subset of these samples has been selected for whole‐gene characterisation. In total, we have characterised and submitted 1056 whole‐gene sequences (Table 2). A total of 898 of the submitted sequences were unique, in part providing the full‐length sequence information for previously named alleles (*n* = 292), in part describing novel alleles (*n* = 606). In cases where we had access to additional samples with identical sequences we submitted these sequences as confirmation (*n* = 158).

As of January 23, 2017, all of the sequences submitted during this project have been named by the World Health Organization (WHO) Nomenclature Committee for factors of the HLA System (Table S1, Supporting information). In 33 of the submitted sequences, a premature stop codon was identified and the corresponding alleles were predicted to be non‐functional (null alleles). In 3 cases, fresh blood samples could be obtained to perform a serological HLA analysis. The alleles could not be detected in any of these cases, supporting their status as null alleles. A total of 12 sequences were submitted with questionable expression status (Q alleles). Those alleles contain characteristic sequences that have been shown in other alleles to affect normal expression. Therefore, 5% of the submitted sequences were characterised as null or Q alleles (Table 3).

**Table 3:** Proportion of null and Q alleles among the submitted sequences

HLA locus	Submitted unique sequences	Null alleles	Q alleles	Total null and Q alleles
A	235	14	4	18 (8%)
B	152	5	0	5 (3%)
C	402	14	6	20 (5%)
DQB1	102	0	2	2 (2%)
DPB1	7	0	0	0 (0%)
Total	898	33	12	45 (5%)

HLA, human leukocyte antigen.

The first 188 of the 1056 alleles were characterised using SBS technology alone. A total of 18 of these alleles, however, were later found in other samples and thereby confirmed. The vast majority of 868 alleles were analysed using the dual redundant sequencing strategy followed by submission with TypeLoader. The full‐length characterisation of novel alleles is an ongoing process where many of the identified novel alleles have yet to be subjected to the dual redundant reference sequencing strategy. In particular, we have not yet targeted class II loci extensively.

### 3.4 Effect on the IPD‐IMGT/HLA Database

**Figure 4:**
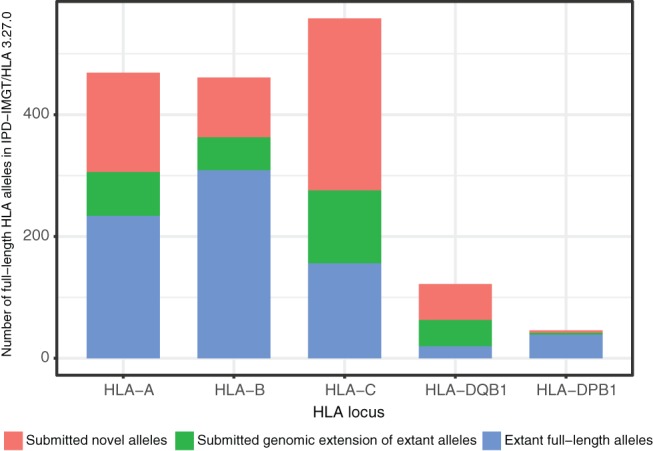
Effects on the IPD‐IMGT/HLA Database. The number of fully characterised human leukocyte antigen (HLA) alleles including the 5′‐ and 3′‐UTR in the IPD‐IMGT/HLA Database release 3.27.0 was more than doubled after the submission of 898 unique full‐length sequences (submitted novel alleles [red], genomic extension of extant allele sequences [green]. Extant fully characterised HLA alleles in IPD‐IMGT/HLA Database release 3.27.0 [blue])

In the IPD‐IMGT/HLA Database release 3.24.0 (April 2016), only 728 (5.2%) of all known HLA alleles for the 6 classical HLA genes were fully characterised. The vast majority of the novel sequences described here have gradually been submitted since this release, and are fully included in the current IPD‐IMGT/HLA Database release 3.27.0 (January 2017). This submission of 898 complete sequences has helped to raise the proportion of fully characterised alleles in the IPD‐IMGT/HLA Database to 10.8% (1697 alleles) and has more than doubled the number of fully characterised alleles in the database (Table 4 and Figure 4).

**Table 4:** Effect of the submitted dataset on the number of fully characterised alleles in the IPD‐IMGT/HLA Database release 3.27.0

HLA locus	IPD‐IMGT/HLA Database release 3.27.0		Submitted fully characterised alleles^a^
	All described alleles	All fully characterised alleles^a^	
A	3830	469 (12.2%)	235 (6.1%)
B	4646	461 (9.9%)	152 (3.3%)
C	3382	558 (16.5%)	402 (11.9%)
DRB1	2010	41 (2.0%)	0 (0%)
DQB1	1054	122 (11.6%)	102 (9.7%)
DPB1	740	46 (6.2%)	7 (0.9%)
Total	15 662	1697 (10.8%)	898 (5.7%)

HLA, human leukocyte antigen.

a Including 5′ and 3′UTR.

### 3.5 Confirmation by routine typing

Many of the alleles discussed above have been named only recently by the WHO Nomenclature Committee for factors of the HLA System. However, the first full‐length HLA alleles of this project, submitted in April 2014, were included in the IPD‐IMGT/HLA Database release 3.17.0 in June, 2014 (Table S1). Since then, DKMS Life Science Lab has genotyped 2.2 million samples and confirmed 27 distinct alleles 196 times in independent samples. Table 5 lists all the confirmed alleles and their respective frequencies in the DKMS donor pool. The frequency is calculated based on the total number of samples that have been genotyped since the allele was named and released. We have not reanalysed the entire donor pool with respect to the latest IPD‐IMGT/HLA release to calculate the exact frequencies based on the total number of 2.7 million samples analysed by NGS. However, this limited analysis already indicates that at least some of these novel alleles are not rare.

**Table 5:** Frequency of novel alleles submitted during this project among DKMS samples

HLA allele	Observations^a^	Total samples^b^ (Mio)	Frequency (%)
DPB1*04:01:31	82	0.6	6.4 × 10^−5^
DPB1*463:01	62	0.6	4.8 × 10^−5^
B*07:252	11	1.3	4.1 × 10^−6^
C*02:92N	6	1.4	2.2 × 10^−6^
DPB1*398:01	4	1.3	1.5 × 10^−6^
B*07:255	3	1.3	1.1 × 10^−6^
C*02:91	3	1.4	1.1 × 10^−6^
C*05:111	3	1.4	1.1 × 10^−6^
C*07:391	3	2.0	7.4 × 10^−7^
C*04:188	2	1.4	7.4 × 10^−7^
B*07:02:48	1	0.7	7.4 × 10^−7^
B*07:237	1	1.3	3.7 × 10^−7^
B*35:01:45	1	1.3	3.7 × 10^−7^
B*49:38	1	0.7	7.2 × 10^−7^
C*02:85	1	2.0	2.5 × 10^−7^
C*02:86	1	2.0	2.5 × 10^−7^
C*04:01:66	1	1.4	3.7 × 10^−7^
C*04:177	1	2.0	2.5 × 10^−7^
C*05:118	1	0.7	7.2 × 10^−7^
C*06:154	1	0.7	7.2 × 10^−7^
C*07:01:45	1	2.0	2.5 × 10^−7^
C*07:02:69	1	0.7	7.2 × 10^−7^
C*07:384	1	2.0	2.5 × 10^−7^
C*07:420	1	0.7	7.2 × 10^−7^
C*12:141	1	1.4	3.7 × 10^−7^
C*12:155Q	1	0.7	7.2 × 10^−7^
C*14:69	1	1.4	3.7 × 10^−7^

a Enquiry date: August 30, 2016.

b Depending on the time of submission and incorporation into the IPD‐IMGT/HLA Database, the total number of samples differs.

## 4 DISCUSSION

The hyperpolymorphic nature of the HLA family of genes and the presence of a large proportion of partially characterised HLA alleles in the IPD‐IMGT/HLA Database present a hurdle to fully exploit the potential of NGS for stem cell transplantation matching. As mentioned, this creates an inherent bias towards the less characterised alleles potentially leading to false allele assignments. Therefore, and given the sequencing capabilities of state‐of‐the‐art NGS instruments, it would be desirable to clearly focus the submission process on full‐length characterised alleles.

Until recently, Sanger sequencing was the preferred method to generate full‐length HLA allele sequences and continues to be in wide use. Because Sanger sequencing inherently lacks any phasing information, a cloning step or allele‐group‐specific PCR are required to separate the alleles in heterozygous samples. Although Sanger sequencing generates highly reliable sequences for fragments in the order of 800 bp, longer sequences require the setup of multiple overlapping sequencing reactions. To avoid sequencing errors, these reactions are commonly performed in both directions to better cope with strand‐specific sequencing artefacts. Therefore, full‐length characterisation of a DPB1 allele would require the setup and analysis of about 40 sequencing reactions. In contrast, using our dual redundant sequencing strategy, 96 DPB1 alleles can be characterised following PCR and library preparation on 1 MiSeq and 2 SMRT sequencing runs. This strategy delivers submission‐grade full‐length sequences at a fraction of the time and cost of Sanger sequencing.

While sequencing on the Illumina HiSeq/MiSeq platforms provides sufficient accuracy to be able to resolve the hyperpolymorphisms referred to earlier, fragment size limitation proves to be a bottle neck for accurately phasing full‐length allele sequences in particular of the long HLA class II loci. In contrast, the PacBio RS II platform, while capable of addressing the phasing issue, lacks the accuracy required for reference sequencing in homopolymer regions owing to a relatively high insertion/deletion sequencing error rate.^14^, ^17^

We developed and implemented a dual redundant sequencing strategy to exploit the individual advantages of the Illumina and Pacific Biosciences instruments while minimising their individual shortcomings by comparing, contrasting and combining the sequences generated from both platforms. An advantage of this strategy is the extremely low probability of the same PCR artefacts occurring twice in 2 different and independent approaches.

Our workflow delivers a large number of highly accurate full‐length allele sequences in a cost‐effective and efficient manner. Subsequently, these sequences need to be submitted to the EMBL‐ENA and the IPD‐IMGT/HLA Database. Given that both the EMBL‐ENA and the IPD‐IMGT/HLA Database require the submission of sequences along with gene model annotation in specific machine readable formats, the manual process of submitting is error‐prone and time‐consuming. We developed TypeLoader to address these issues.^15^ Using basic file formats (XML or FASTA), TypeLoader creates all the required files for those processes automatically and features seamless submission to the IPD‐IMGT/HLA Database repository. It automates almost all manual steps involved in this procedure and reduces the time spent on manual curation by more than 95%.

To fully exploit the potential of NGS for stem cell transplantation matching, the existing gaps in the IPD‐IMGT/HLA Database need to be closed. The adaptation of both, the Illumina and PacBio platforms in standard sequencing workflows open up the opportunity to expand the IPD‐IMGT/HLA Database repository comprehensively. We have developed this dual redundant sequencing strategy with the goal of contributing complete reference sequences to the genotyping sequence repositories as much as possible.

## Supporting information


**Table S1.** List of alleles and samples for which full length sequences were submitted.
